# Correction: Zavestovskaya et al. Boron Nanoparticle-Enhanced Proton Therapy for Cancer Treatment. *Nanomaterials* 2023, *13*, 2167

**DOI:** 10.3390/nano15211640

**Published:** 2025-10-28

**Authors:** Irina N. Zavestovskaya, Anton L. Popov, Danil D. Kolmanovich, Gleb V. Tikhonowski, Andrei I. Pastukhov, Maxim S. Savinov, Pavel V. Shakhov, Julia S. Babkova, Anton A. Popov, Ivan V. Zelepukin, Maria S. Grigoryeva, Alexander E. Shemyakov, Sergey M. Klimentov, Vladimir A. Ryabov, Paras N. Prasad, Sergey M. Deyev, Andrei V. Kabashin

**Affiliations:** 1 P. N. Lebedev Physical Institute of the Russian Academy of Sciences, Leninsky Prospect 53, 119991 Moscow, Russia; a.popov@lebedev.ru (A.L.P.); kdd100996@mail.ru (D.D.K.); grigorevams@lebedev.ru (M.S.G.); shemyakovae@lebedev.ru (A.E.S.); ryabov@lebedev.ru (V.A.R.); 2 Bionanophotonics Laboratory, Institute of Engineering Physics for Biomedicine (PhysBio), National Research Nuclear University MEPhI (Moscow Engineering Physics Institute), Kashirskoe Shosse 31, 115409 Moscow, Russia; gvtikhonovskii@mephi.ru (G.V.T.); mssavinov@mephi.ru (M.S.S.); pvshakhov@mephi.ru (P.V.S.); babkovaserg@gmail.com (J.S.B.); aapopov1@mephi.ru (A.A.P.); smklimentov@mephi.ru (S.M.K.); pnprasad@buffalo.edu (P.N.P.); biomem@mail.ru (S.M.D.); 3 Institute of Theoretical and Experimental Biophysics, Russian Academy of Sciences, 3 Institutskaya St., 142290 Pushchino, Russia; 4 LP3, Aix-Marseille University, CNRS, 13288 Marseille, France; andrei.pastukhov@etu.univ-amu.fr; 5 Shemyakin-Ovchinnikov Institute of Bioorganic Chemistry, Russian Academy of Sciences, 117997 Moscow, Russia; ivan.zelepukin@gmail.com; 6 Department of Chemistry, Institute for Lasers, Photonics, and Biophotonics, The State University of New York at Buffalo, Buffalo, NY 14260, USA; 7 “Biomarker” Research Laboratory, Institute of Fundamental Medicine and Biology, Kazan Federal University, 18 Kremlyovskaya St., 420008 Kazan, Russia; 8 Institute of Molecular Theranostics, Sechenov First Moscow State Medical University, 119991 Moscow, Russia

In the original publication [1], there was a mistake in Figure S2 as published. Two of the sub-images in Figure S2 were duplicates. The corrected Figure S2 appears below. The authors state that the scientific conclusions are unaffected. This correction was approved by the Academic Editor. The original publication has also been updated.

## Figures and Tables

**Figure S2:**
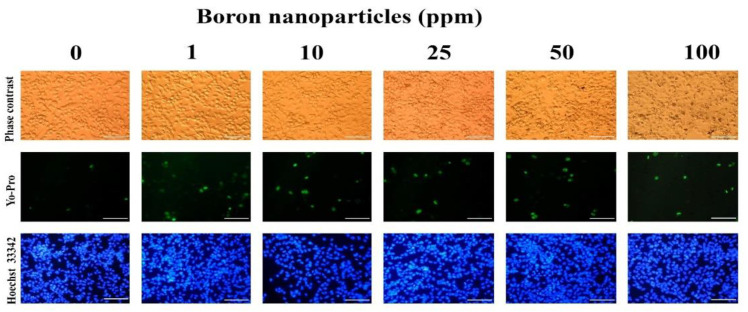
Analysis of cell morphology, apoptosis and Live/Dead assay 24 h after incubation with boron nano-particles. Scale bar—100 μm.
