# Supplementary material for: Boron Nanoparticle-Enhanced Proton Therapy for Cancer Treatment
Source: Nanomaterials (Basel). 2023 Jul 26;13(15):2167. doi: 10.3390/nano13152167 (PMC10421420; doi:10.3390/nano13152167)
Supplement: Supplementary file 1 [file nanomaterials-13-02167-s001.zip › nanomaterials-2482687-supplementary.pdf]

Supplementary materials

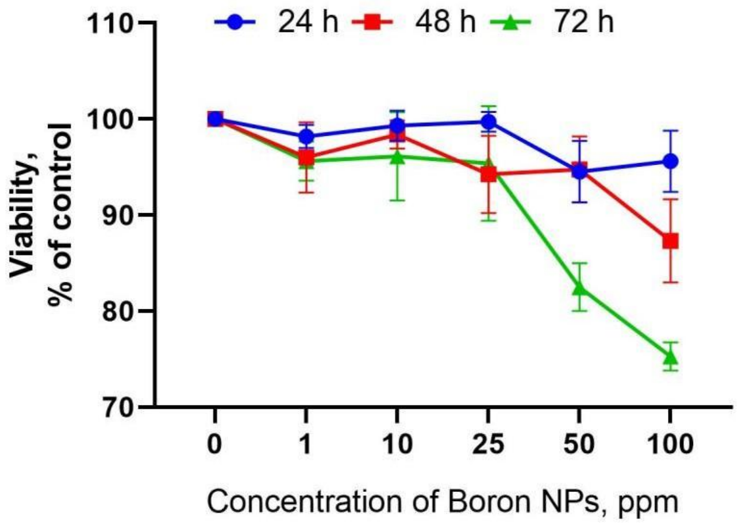

Figure S1. Cell viability via MTT assay after 24, 48 and 72 h of incubation with boron nanoparticles.

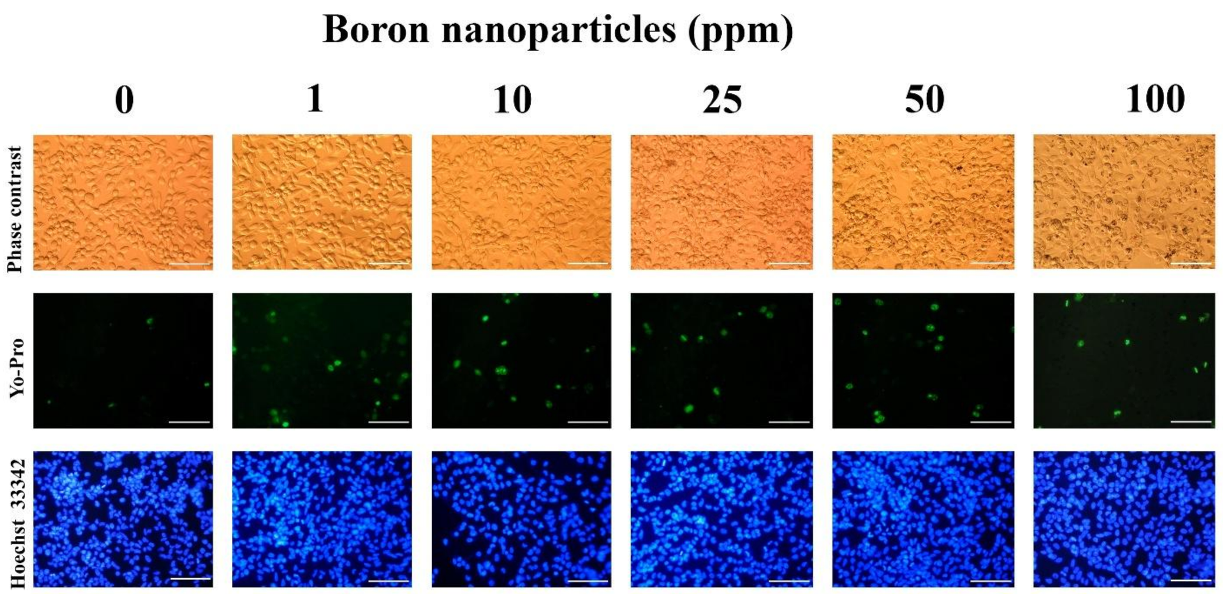

Figure S2. Analysis of cell morphology, apoptosis and Live/Dead assay 24 h after incubation with boron nanoparticles. Scale bar—100 μm.

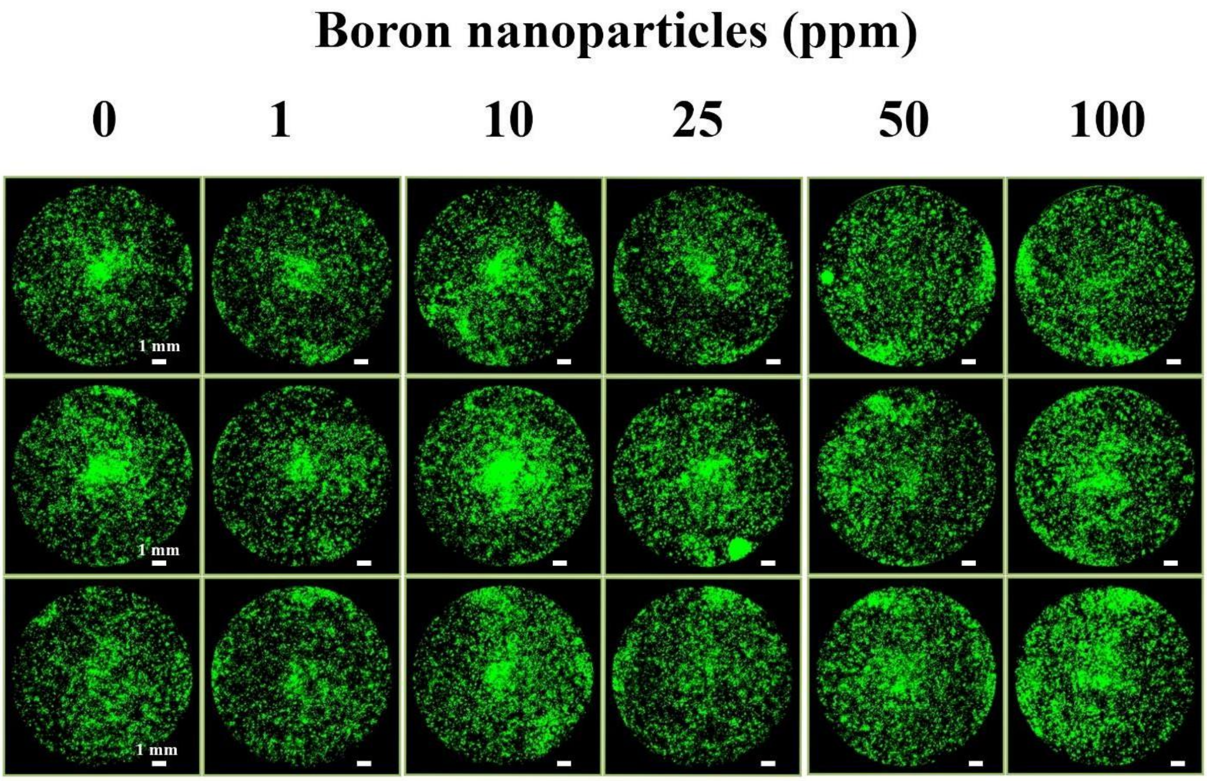

Figure S3. Clonogenic assay after 24, 48 and 72 h of incubation with boron nanoparticles. Scale bar—1 mm.

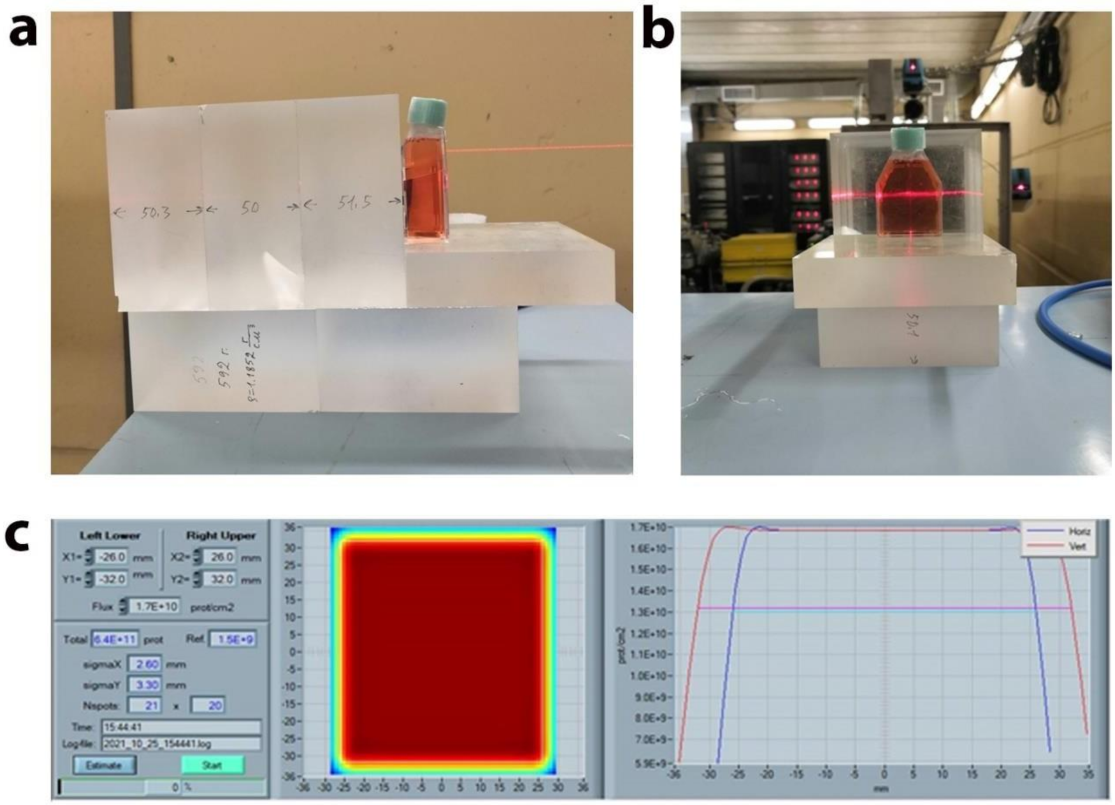

Figure S4. Scheme of irradiation of culture flasks (**a**,**b**) and dose distribution in the module for calculating the irradiation zone with a graph of dose distribution along the central horizontal and vertical axes (**c**).

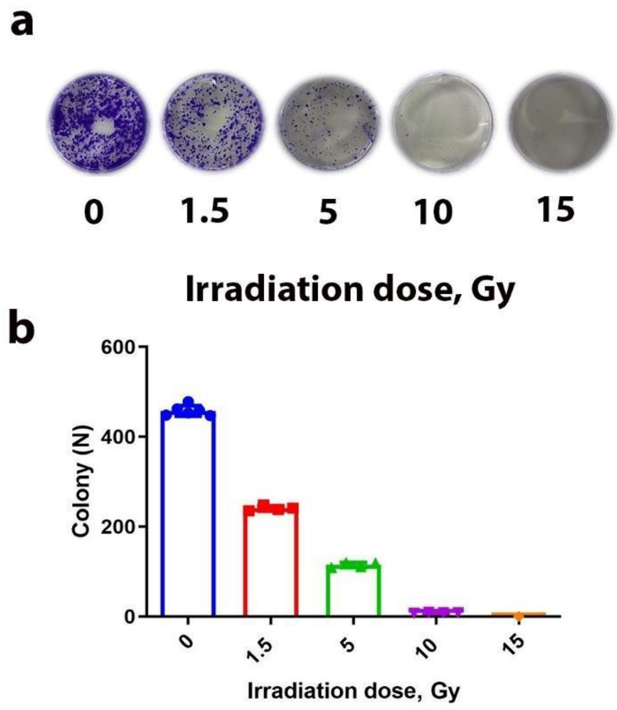

Figure S5. Dose-dependent response of clonogenic activity after proton beam irradiation in the Bragg peak.
